# Visual Calibration for Multiview Laser Doppler Speed Sensing

**DOI:** 10.3390/s19030582

**Published:** 2019-01-30

**Authors:** Yunpu Hu, Leo Miyashita, Yoshihiro Watanabe, Masatoshi Ishikawa

**Affiliations:** 1 Department of Creative Informatics, The University of Tokyo, 7-3-1 Hongo, Bunkyo-ku, Tokyo 113-8656, Japan; leo_miyashita@ipc.i.u-tokyo.ac.jp (L.M.); masatoshi_ishikawa@ipc.i.u-tokyo.ac.jp (M.I.); 2 Department of Information and Communications Engineering, School of Engineering, Tokyo Institute of Technology, 4259-G2-31, Nagatsuta, Midori-ku, Yokohama, Kanagawa 226-8502, Japan; watanabe.y.cl@m.titech.ac.jp

**Keywords:** calibration, motion sensing, laser doppler velocimetry, computer vision, optimization

## Abstract

We present a novel calibration method for a multi-view laser Doppler speed sensing (MLDSS) system. In contrast with the traditional method where only the laser geometry is independently calibrated, the proposed method simultaneously optimizes all the laser parameters and directly associates the parameters with a motion sensing model. By jointly considering the consistency among laser Doppler velocimetry, the laser geometry and a visual marker tracking system, the proposed calibration method further boosts the accuracy of MLDSS. We analyzed the factors influencing the precision, and quantitatively evaluated the efficiency of the proposed method on several data sets.

## 1. Introduction

Multiview laser Doppler speed sensing (MLDSS) [1] uses several one-dimensional speed measurements to recover the six-degree-of-freedom (6-DOF) motion of an arbitrary rigid body. Such a system has unique advantages, such as the capability to measure untextured and unstructured targets, ultrahigh speed, and ultralow computational cost. An inertial measurement unit [2,3] may have similar characteristics, but would be intrusive since it must be attached to the target. On the other hand, conventional contactless 6-DOF motion-sensing techniques, including computer-vision approaches, usually rely on the target structure [4,5] and texture [6,7], making it difficult to directly reapply them when the scenario changes. In this sense, MLDSS is potentially a more general solution for most motion-sensing tasks, e.g., industrial inspection, user interface [8], and autonomous driving.

It is important to mention that the precision of MLDSS strongly relies on the sensing accuracy of the laser Doppler velocimeter (LDV), the system-layout parameters, and calibration precision. Factors related to the LDV sensing, such as speckle noise [9,10] and laser source drift [11,12] have been well discussed. Hu et al. stated the essential system-layout conditions, and proposed a simple method to calibrate the system parameters [1]. In their approach, each laser was separately calibrated by linearly fitting of the camera-collected 3D laser spot co-ordinates.

A similar calibration principle can be commonly found in the literature for the calibration of galvanoscopic laser systems using various types of sensors [13,14,15]. It makes sense and achieves relatively good accuracy, but it has to be noted that LDV measurements and the system layout are not involved in the process. Hence, we refer to it as “geometric-only calibration”. Although measurement precision is rather sensitive to the accuracy of the calibration parameters, geometric-only calibration minimizes only the error in image-based measurements, but ignores correlation with the motion-reconstruction process.

The inspiration for our approach is derived from the calibration techniques of optical systems and computer vision, in which simultaneously calibrating all elements is rather beneficial in terms of both simplicity and accuracy [16,17]. It is also important to incorporate a system measurement model into the geometrical calibration, as implied in References [18,19]. Based on this idea, we present a novel calibration technique for MLDSS. This is done with a maximum-likelihood estimate (MLE) for the system parameters given the measurements of the LDV, and the motion ground truth provided by an additional 3D tracking vision system. Instead of directly calculating the laser geometry, in this method we sought the best group of laser parameters that minimized motion-estimation error. We validated the proposed method on several different datasets, and an obvious improvement in accuracy was confirmed.

## 2. MLDSS Parameters

In this section, we briefly describe the basic kinematic formulation in MLDSS [1] and parameterize it for the calibration.

### Basic Equations

We discuss a right-handed co-ordinate system with a fixed origin O. A rigid body has a 6-DOF speed denoted by $X={\left[\omega^{T},v^{T}\right]}^{T}$, with angular components $\omega ={\left[\omega_{x},\omega_{y},\omega_{z}\right]}^{T}$ and linear components $v={\left[v_{x},v_{y},v_{z}\right]}^{T}$.

MLDSS utilizes a simple kinematic model to reconstruct the speed of a target rigid body. When a laser-beam ray hits the target, the one-dimensional velocity along this ray is measured by the Doppler effect. Let the $i_{th}$ laser be denoted by $L_{i}:o_{i},l_{i}$, where $o_{i}$ is one point on $L_{i}$, and $l_{i}$ is the direction unit vector. Velocity measurement could then be formulated as:

(1) $$v_{i}=o_{i}\times l_{i}\cdot \omega +l_{i}\cdot v$$

**Figure 1:**
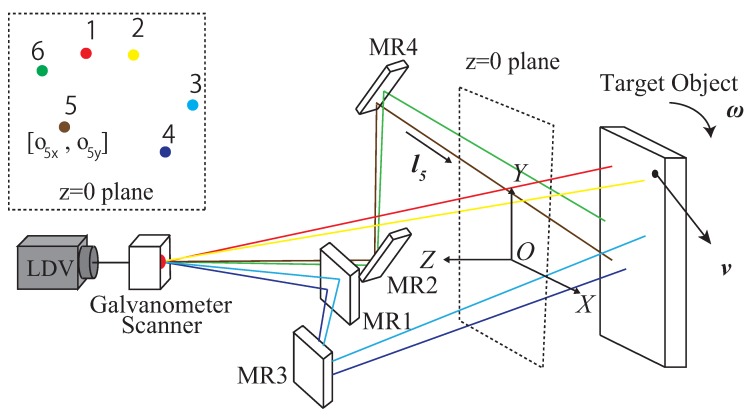
System to be calibrated and its parameters. Laser beams emitted from the laser Doppler velocimeter (LDV) are reflected by the galvanometer scanner and mirror array MR1~MR4, and finally hit a moving-target object. By scanning the laser on the target surface, the six-degree-of-freedom (6-DOF) speed of the target could be reconstructed. Parameters consist of laser geometry, denoted by the x and y co-ordinates on the z = 0 plane, and the laser-direction angles.

By combining multiple measurements with the form in Equation (1), the 6-DOF speed could be linearly reconstructed from one-dimensional velocity measurements $v_{i}$. The minimum MLDSS implementation with six lasers, as shown in Figure 1, is written as:

(2) $$\left[\begin{array}{cc} {\left(o_{1}\times l_{1}\right)}^{T} & l_{1}^{T}\\ {\left(o_{2}\times l_{2}\right)}^{T} & l_{2}^{T}\\ \vdots & \vdots \\ {\left(o_{6}\times l_{6}\right)}^{T} & l_{6}^{T} \end{array}\right]\left[\begin{array}{c} \omega \\ v \end{array}\right]=\left[\begin{array}{c} v_{1}\\ v_{2}\\ \vdots \\ v_{6} \end{array}\right]$$

We use *A* to denote the leftmost matrix, and b to denote the rightmost vector in Equation (2). Matrix *A* fully describes the system, but the dimension of the parameter space is smaller than the number of matrix elements. Consider the $i_{th}$ row of *A*, ${\left(A\right)}_{i}$. It represents the measurement process of laser $L_{i}$, determined by $o_{i}$ and $l_{i}$. Note that $o_{i}$ is a point on a straight line, and $l_{i}$ is a unit vector. Both $o_{i}$ and $l_{i}$ have only two DOFs, so that ${\left(A\right)}_{i}$ has four DOFs. Under the optical system layout and the specified co-ordinate system illustrated in Figure 1, we let $o_{i}={\left[o_{ix},\;o_{iy},\;0\right]}^{T}$ and $l_{i}=\left[\cos\beta_{i}\cos\alpha_{i},\sin\beta_{i}\cos\alpha_{i},\sin\alpha_{i}\right]$, without loss of generality. Then, the parameters of the system could be denoted by

(3) $$P=\{o_{ix},o_{iy},\alpha_{i},\beta_{i}|i=1,2\ldots 6\}$$

where *P* consists of 24 elements.

## 3. MLDSS Calibration

### 3.1. Geometric-Only Calibration

Note that *P* consists of only the geometrical parameters of the lasers. We can directly calculate each laser’s spatial position, i.e., $o_{i},l_{i}$, in the calibration.

**Figure 2:**
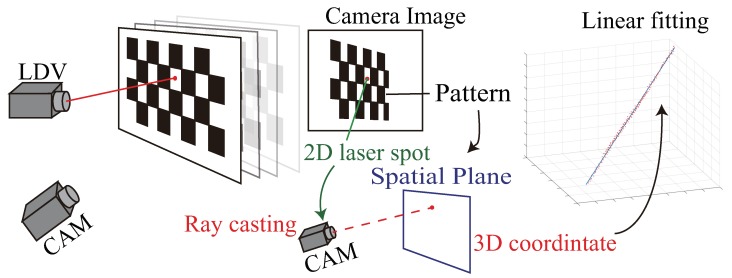
Geometric-only calibration procedures.

We first briefly describe this geometric-only calibration method [1,14,15]. As illustrated in Figure 2, a planar board with printed pattern such as a chessboard is used as the calibration object. When the laser hits the object, both the pattern and the laser spot are captured by a camera. With no loss of generality, we assume the camera is already calibrated. The position of the pattern can be calculated from the image [20], and the 3D co-ordinates of the laser spot can be computed by casting a ray from the camera to the pattern plane. This procedure is repeated, and the laser parameters are acquired by linear fitting of multiple 3D point co-ordinates. Note that a similar principle can be used with a different sensor, such as a depth camera [13].

Apparently, this method has geometrical errors from sources such as the image processing error and lens distortion. Here, we go one step farther to see the error of motion reconstruction. Consider Equation (2). We can split matrix *A* into a ground-truth matrix $A_{g}$ and a distortion matrix $A_{\delta }$. The motion reconstruction error related to the calibration error is then denoted by $\delta X\approx A_{g}^{-1}A_{\delta }X$. Comparing with the error introduced by the LDV measurement denoted by $\delta X=A^{-1}\delta b$, the calibration error introduces an unbounded error that linearly increases when target speed X becomes larger. Hence, in the case of sensing large speed, calibration accuracy could be much more important than LDV measurement accuracy. However, the geometric-only methods only minimize the geometric error (e.g., reprojection error), so that the motion-reconstruction error is prone to larger target speed and systematic errors of the calibration system.

The intuition here, as we claimed in Section 1, is that all laser parameters should be jointly optimized, and motion-reconstruction error should be minimized instead of laser geometry error, so that bounded motion-reconstruction accuracy could be expected.

### 3.2. Statistical Calibration by Minimizing Motion-Reconstruction Error

Hence, in this paper, the calibration of MLDSS is described as how to find the best set of parameters *P* that generate the most accurate 6-DOF speed estimation $\tilde{X}$ under the current system layout, given LDV measurements b.

To simplify the discussion, we divided motion sensing into discrete frames. First, assume that we know the ground-truth position of the target object at each frame *k*. It is denoted by $\xi_{k}={\left[r_{k}^{T},t_{k}^{T}\right]}^{T}$ in the space of se(3) Lie algebra, where $r_{k}={\left[r_{kx},r_{ky},r_{kz}\right]}^{T}$ is the orientation and $t_{k}={\left[t_{kx},t_{ky},t_{kz}\right]}^{T}$ is the location. Simultaneously, the MLDSS system measures velocities $b_{k}={\left[v_{k1},v_{k2},v_{k3},v_{k4},v_{k5},v_{k6}\right]}^{T}$, respectively from each laser.

Note that long-term speed integration would inevitably introduce larger and time-dependent error, a short-time interval may result in undesired peaks in the estimated speed, and interframe subtraction helps to cancel systematic errors in a 3D tracking system. We formulate the problem with interframe motion increments. The pose change between frame *k* and k−1 is thus represented by $\delta \xi \left(k\right)=\left[\delta r_{k}^{T},\delta t_{k}^{T}\right]$, where

(4) $$\begin{array}{cc} \delta r_{k} & =r_{k}-r_{k-1}\\ \delta t_{k} & =t_{k}-exp\left(\delta r_{k-1}\right)t_{k-1} \end{array}$$

exp(·) denotes the conversion from a so(3) rotation vector to the corresponding SO(3) rotation matrix, known as the Rodrigues transform [21].

Knowing that matrix *A* can be uniquely determined by parameter set *P*, the 6-DOF speed of the target measured by the MLDSS system at frame *k* could be calculated by ${\tilde{X}}_{k}\left(P\right)={\left[A\left(P\right)\right]}^{-1}b_{k}$. Let $\delta t_{k}=t_{k}-t_{k-1}$. The interframe pose-change estimation of the MLDSS system is thus written as

(5) $$\delta {\tilde{\xi }}_{k}\left(P\right)={\tilde{X}}_{k}\left(P\right)\delta t_{k}$$

We use $e_{k}\left(P\right)=\delta \xi_{k}-\delta {\tilde{\xi }}_{k}\left(P\right)$ to denote the error of one frame. With the assumption that $\delta {\tilde{\xi }}_{k}\left(P\right)$ is locally linear, and the LDV measurement error follows a Gaussian distribution, the MLE of the MLDSS system is the solution of the following nonlinear least-square problem:

(6) $$P_{opt}=\arg\min_{P}\sum_{k}\left(e_{k}^{T}\left(P\right)e_{k}\left(P\right)\right))$$

We solve Equation (6) using the Levenberg–Marquardt algorithm [22]. It requires an initial guess of *P*, which can be provided by the geometric-only method as introduced in Section 3.1.

### 3.3. System Setup and Nonideal Factors

It has to be mentioned that the description in Section 3.2 omitted some details in order to clearly state the proposed method. In this subsection, we describe the practical system for calibration and reformulate the complete calibration problem.

**Figure 3:**
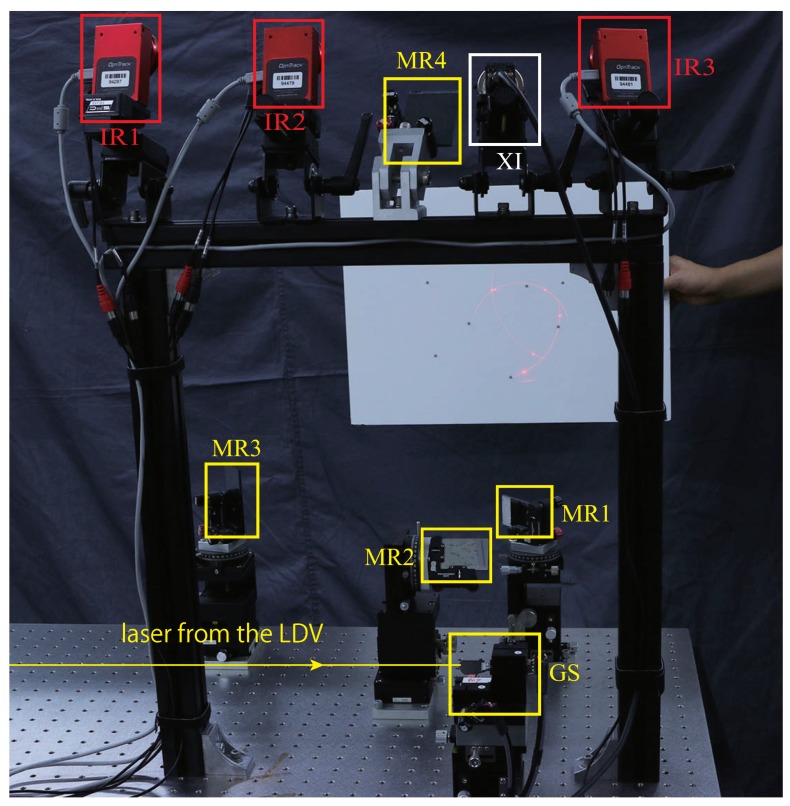
Experimental setup. Photograph of the system and the experimental condition. MR1~MR4 are the mirrors, and GS is the galvanometer scanner. IR1~IR3 are IR illumination sources and cameras. XI is a monochrome camera. Calibration object was moved manually 1 m away from the system.

Figure 3 illustrates the system for calibration. It consists of the MLDSS system to be calibrated [1], a marker-based 3D tracking system composed of three IR cameras (Flex 3, Natural Point, Inc., Corvallis, OR, USA), and a monochrome camera (MQ003MG-CM, XIMEA). The 3D tracking system uses active infrared illumination and the stereo method to track the 3D location of retroreflective markers. Four or more asymmetrically placed markers on a rigid body can be tracked, with the 6-DOF pose denoted by Lie Algebra ξ. This pose is taken as the ground truth in our discussion, since most of the systematic error is cancelled by interframe subtraction.

The four cameras were calibrated [23,24] beforehand. The IR camera’s trigger signal was inverted each time the LDV measurement was sampled, so that the frame rate of the MLDSS was roughly twice that of the cameras. Throughout the discussion in this paper, the MLDSS system ran at about 200 Hz, and the tracking system ran at 100 Hz. All devices were controlled by a desktop workstation (Dell T7910 workstation with Intel E5-2687W v4 @3GHz). Note that such a high-spec computer is not necessary for this calibration. Both motion reconstruction and optimization in this paper are computationally friendly and can run on common tabletop computers.

We address the nonideal factors in this practical calibration system as follows.

**Temporal misalignment.** As we described above, the 3D tracking system runs slower than the MLDSS system so that they are temporally misaligned. Using *f* to denote the frame index of MLDSS, and $t_{f}$ to denote the corresponding time, we align the MLDSS measurements to the 3D tracking system. Equation (5) is thus reformulated by linearized integration and interpolation:

(7) $$\delta {\tilde{\xi }}_{k}\left(P\right)=\sum_{f}{\tilde{X}}_{f}\left(P\right)\delta t_{f}$$

where

(8) $$\begin{array}{c} \delta t_{f}=min\left(t_{f}-t_{k-1},t_{f}-t_{f-1},t_{k}-t_{f}\right)\\ t_{k-1}\leq t_{f}\leq t_{k} \end{array}$$

Note that although the two systems are synchronized by hardware connection, unintended temporal misalignment might still exist due to the sampling time of the LDV and the exposure time of the cameras. This misalignment causes an error in $e_{k}\left(P\right)$ and is independent of parameters *P* that finally influence calibration precision. To solve it, we modeled the temporal misalignment as a fixed offset parameter $t_{o}$. Equation (8) is thus rewritten as

(9) $$\begin{array}{c} \delta t_{f}=min\left(t_{f}-t_{k-1}+t_{o},t_{f}-t_{f-1},t_{k}-t_{f}-t_{o}\right)\\ t_{k-1}-t_{o}\leq t_{f}\leq t_{k}-t_{o} \end{array}$$

**Mechanical velocity offset.** A galvanometer scanner is used for controlling laser direction. While we assumed the scanner fully stopped when sampling the LDV measurement, the scanner could possibly slightly oscillate and cause error in the speed. Because the control sequence of the galvanometer is fixed and the optical path of each laser is different, this offset can be regarded as systematic to each laser. We thus refined speed reconstruction as

(10) $${\tilde{X}}_{f}\left(P\right)={\left[A\left(P\right)\right]}^{-1}\left(b_{f}+b_{o}\right)$$

where $b_{o}$ is the vector composed of the velocity offset for each laser due to the corresponding mechanical oscillation.

**Weighing error terms.** In solving the multiobject optimization problem denoted by Equation (6), it is important to balance the influence of each error term and data samples in order to not let partial data be dominant. Here, we introduce a diagonal weight matrix *W*. Error term $E_{k}$ for each sample *k* is thus:

(11) $$E_{k}\left(P\right)=e_{k}^{T}\left(P\right)We_{k}\left(P\right)$$

Considering the difference in the unit and the distance from the calibration object to the origin of the system, [1,1,1,0.005,0.005,0.005] is ad hoc determined as the diagonal elements of *W* in our experiment, but note that this weight should be adjusted with a change in the co-ordinate system.

**Outlier Measurements.** Speckle noise [10] is one of the main noise sources of the LDV. It causes undesired short-term peaks in velocity measurements. Such noise can largely be relieved in the low-frequency domain with the use of a tracking filter [1,9]. However, considering signal intensity is low due to potential loss in the laser focus, there occasionally is speckle noise in measurements. In order to reduce its influence on calibration accuracy, a modified Huber kernel [25] is introduced to the problem:

(12) $$E_{k}\left(P\right)=\left\{\begin{array}{cc} \frac{e_{k}^{T}\left(P\right)We_{k}\left(P\right)}{2\delta } & \mathrm{if}\;e_{k}^{T}\left(P\right)We_{k}\left(P\right)<\delta^{2}\\ \sqrt{e_{k}^{T}\left(P\right)We_{k}\left(P\right)}-\frac{\delta }{2} & \mathrm{otherwise}. \end{array}\right.$$

where outlier errors grow linearly instead of quadratically.

**Complete maximum likelihood estimation.** With the above consideration, we can naturally extend Equation (6) by estimating the complete set of parameters:

(13) $$P_{opt}=\arg\min_{P,t_{o},b_{o}}\sum_{k}\left(E_{k}\left(P,t_{o},b_{o}\right)\right)$$

where $E_{k}\left(P,t_{o},b_{o}\right)$ is defined by injecting Equations (7), (9), (10) into Equation (12). Equation (13) is solved with the Levenberg–Marquadt algorithm [22] as well. The initial guess of P is provided by geometric-only calibration. $t_{o}$ and $b_{o}$ are simply set to zero.

### 3.4. Summary

In this section, the calibration of MLDSS with a pratical system is described and formulated. The proposed method states the calibration problem with minimization of the motion error, which is essentially different from conventional geometrical methods [1,14,15]. The procedures of the proposed calibration are summarized as follows:

- Make a calibration object with four or more asymmetric placed markers;
- register the calibration object in the 3D tracking system as a trackable rigid body;
- simultaneously capture target motion and speed with the MLDSS and the 3D tracking system, and build a dataset following the instructions in Section 4;
- estimate initial parameters using the geometric-only method introduced in Section 3.1 (optional); and
- refine all parameters by solving Equation (13).

## 4. Data Collection

It is straightforward to solve Equation (13) with the collected motion data. However, inappropriate data selection would provide insufficient information, and finally result in an ill-conditioned optimization problem. We study the data collection for our method in this section.

Check the derivatives of Equation (2). Jacobian $J_{P}=\partial X/\partial P$ describes the gradient direction in the parameter space of one data sample. Let the parameter of laser $L_{i}$ be denoted by $P_{i}$, and the $i_{th}$ column of $A^{-1}$ be ${\left[A^{-1}\right]}_{i}$. Knowing that *A* is a function of *P*, we write the partial Jacobian $J_{i}$ as:

(14) $$J_{i}=\frac{\partial X}{\partial P_{i}}=-{\left[A^{-1}\right]}_{i}X^{T}G_{i}$$

where $G_{i}$ is given by Equation (15).

(15) $$G_{i}=\frac{\partial {\left(A\right)}_{i}}{\partial P_{i}}=\left[\begin{array}{cccc} 0 & \sin\alpha_{i} & o_{iy}\cos\alpha_{i} & 0\\ -\sin\alpha_{i} & 0 & o_{ix}\cos\alpha_{i} & 0\\ \sin\beta_{i}\cos\alpha_{i} & \cos\beta_{i}\cos\alpha_{i} & o_{iy}\cos\beta_{i}\sin\alpha_{i}-o_{ix}\sin\beta_{i}\sin\alpha_{i} & o_{ix}\cos\beta_{i}\cos\alpha_{i}+o_{iy}\sin\beta_{i}\cos\alpha_{i}\\ 0 & 0 & -\cos\beta_{i}\sin\alpha_{i} & \sin\beta_{i}\sin\alpha_{i}\\ 0 & 0 & -\sin\beta_{i}\sin\alpha_{i} & -\cos\beta_{i}\sin\alpha_{i}\\ 0 & 0 & \cos\alpha_{i} & 0 \end{array}\right]$$

$J_{P}$ is the stack of $J_{i}$ for i=1,2,…,6. It can be observed that some submatrices of $J_{P}$ have only zero elements. It means that a certain kind of motion error would provide no additional constraints for some parameters. Especially from Equation (14), error in the translation components does not provide any constraint for position parameters $o_{ix}$ and $o_{iy}$, and the error of $r_{x}$, $r_{y}$ provides no constraint for direction angle $\beta_{i}$.

More intuition is provided by Table 1, where we simulated the norms of the submatrices of $J_{P}$ using real calibration parameters. Subtle, Rot, and Trans denote the motion patterns of subtle speed $X={\left[0.1,0.1,0.1,0.1,0.1,0.1\right]}^{T}$, a rotational speed where $\omega_{x}=\pi rad/s$, and a translational speed where $v_{x}=100mm/s$, respectively. a and o denote the set of direction parameters $\left\{\alpha_{i},\beta_{i}\right\}$ and position parameters $\left\{o_{ix},o_{iy}\right\}$ for all lasers.

Table 1 demonstrates how the motion pattern influences gradient direction. A large translational motion means a larger gradient in the direction parameters a and a smaller gradient in position parameters o. Jointly consider this with Equation (14). Translational motion error in one direction cannot fully condition the parameters, but the absolute value of the gradient could be much larger than the rotational motion. This might finally result in overfitting to a partial dataset, while the other part of the calibration data contributes little to the optimization.

Two implications could be derived to help such a situation. First, contribution to the optimization problem from different samples should be balanced considering the corresponding gradient. This is done by reweighing error terms as we introduced in Section 3.3. Second, multiple motion patterns should be included and evenly distributed in the dataset in order to avoid degeneration configuration.

**Figure 4:**
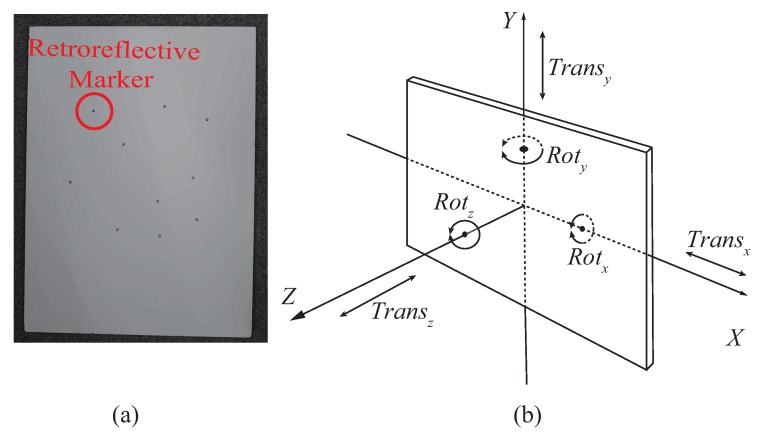
(**a**) Calibration object, a white board with ten randomly but asymmetrically placed retroreflective markers. (**b**) Six motion patterns of the calibration object in its local co-ordinate system, of which the calibration data are composed.

Based on the above discussion, we prepared the dataset for solving Equation (13) with a calibration object as illustrated in Figure 4a In the dataset, the motion of the calibration object was evenly distributed into six different patterns as illustrated in Figure 4b. During data collection, the calibration object was manually moved roughly 1 m away from the measurement systems, as shown in Figure 3.

## 5. Evaluation

### 5.1. Cross-Validation

We validated the proposed method with a real dataset, consisting of 4500 sample frames for the calibration, and 1500 frames for the test. Both the calibration set and the test set consisted of six evenly distributed back-and-forward motion patterns, as shown in Figure 4b. This data amount far exceeds the necessary amount for the calibration, but it helps to reduce data noise. Note that the proposed system runs in high speed. Specifically, the marker tracking system runs at 100 Hz, and the MLDSS runs at 200 Hz. Hence, it takes no more than 10 min to collect all the data and finish optimization.

We compared the proposed method with the geometric-only calibration method [1], where the camera-calibration reprojection error was below 0.081 pixel, and the 3D point-to-line root mean square (RMS) distance in the linear fitting was 0.734 mm (100 points for each laser). Note this error is already smaller than that of the state-of-art geometric-only method [13] thanks to the use of a long-focus camera lens. Since in the MLDSS scenario we care most about motion reconstruction, while geometrical error is of minor importance, we compared the correctness of the reconstructed-motion increments in the test set.

**Figure 5:**
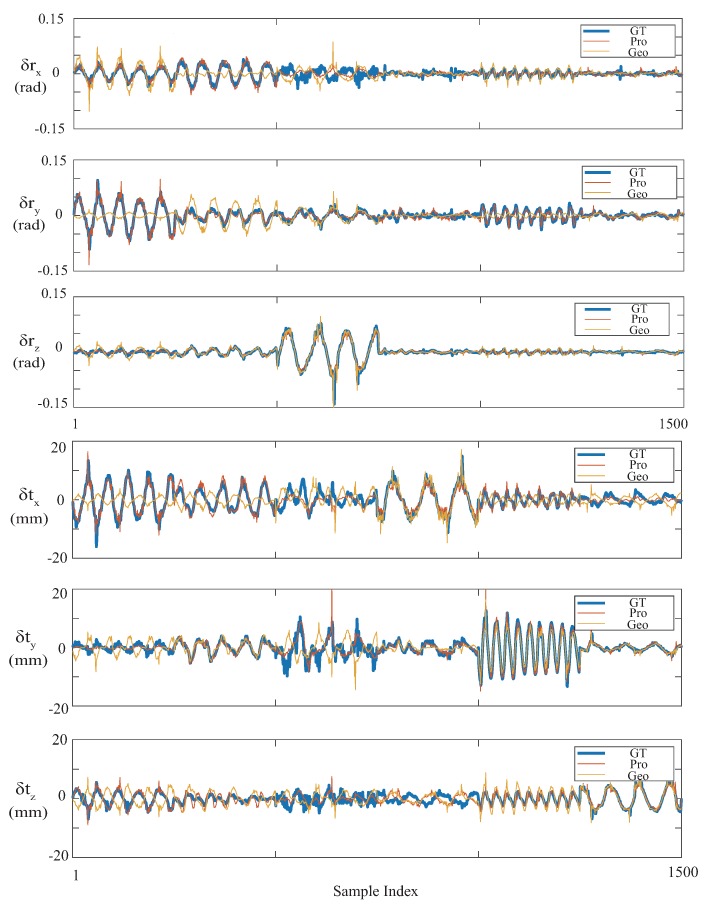
Motion reconstruction results of the test set. Specifically, this figure includes the rotational components (top three subfigures) and translational components (bottom three subfigures) of the interframe motion increments in the test set. It was confirmed that the proposed method obviously outperforms the geometric-only method.

Figure 5 illustrates the six motion components in the reconstruction results of all the samples in the test set. We qualitatively confirmed that motion reconstruction was improved with the proposed method in this figure. While the geometric-only method achieved relatively good accuracy in major motion components, such as in the translational motion, the capability of measuring subtle and rotational motions degraded. With Equation (14), we consider the reason for this as the error in the wrong estimation of positional parameters $o_{ix}$ and $o_{iy}$, and a joint effect of geometrical errors of multiple lasers. In contrast, the proposed method achieved better accuracy in almost all motion components, as shown in the magnified parts of the figure.

To quantitatively demonstrate the results, RMS error between the motion-reconstruction result and the ground truth is shown in Table 2. We divided the test data into six parts by major motion pattern, as demonstrated in Figure 4b. From Table 2, it is shown that the proposed method achieved smaller motion-reconstruction error in almost all motion patterns. The RMS errors of both the rotational and translational components across the whole dataset were reduced by more than 50%, which we consider significant improvement of accuracy.

### 5.2. Sensing Daily Object

**Figure 6:**
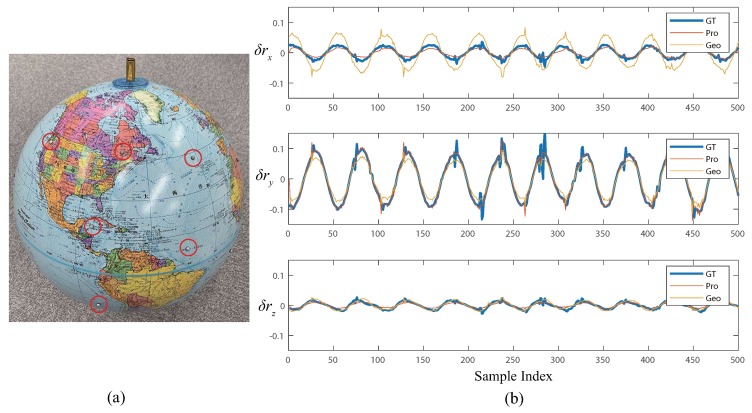
(**a**) Used test globe. Retroreflective markers (red-circled) were attached on the globe to track its rotation. (**b**) Motion-reconstruction results (rotational part).

To show that the proposed method is also valid for the speed estimation of daily objects besides the calibration board, we briefly tested the new parameters on measuring a rotating globe as shown in Figure 6a. Ground-truth motion was also measured by the 3D marker tracking system. Figure 6b shows the result. The proposed method achieved a more accurate result than the geometric-only method. Rotational RMS error was 0.0245 rad with the proposed calibration parameters, while that of the conventional method was 0.0388 rad.

## 6. Conclusions

In this paper, we described a new calibration method for MLDSS. In contrast with the conventional method where only laser geometry is independently calibrated from images, the proposed method simultaneously optimizes all laser parameters and directly associates them with the motion-sensing model. We discussed in detail the system model and factors related to calibration accuracy, and proposed a maximum-likelihood estimation-based method for solving a calibration problem. Finally, higher accuracy was confirmed by cross-validation from qualitative and quantitative evaluation on a 1500 sample test set compared with the state-of-art method.

## Figures and Tables

**Table 1:** *∞*-norm of submatrices under different motions.

Motion Pattern	${∥\partial \omega /\partial a∥}_{\infty }$	${∥\partial \omega /\partial o∥}_{\infty }$	${∥\partial v/\partial a∥}_{\infty }$	${∥\partial v/\partial o∥}_{\infty }$
Subtle	0.0036	0.0012	0.280	0.2201
Rot	0.0088	0.0424	0.501	3.39
Trans	1.31	0	99.2	0

**Table 2:** Root mean square (RMS) errors in the test data.

Test Set	Rotational (Rad)		Translational (mm)	
	Proposed	Geo	Proposed	Geo
$Rot_{x}$	0.0139	0.0484	2.3142	9.0864
$Rot_{y}$	0.0108	0.0321	2.0543	4.9750
$Rot_{z}$	0.0239	0.0321	3.8263	7.6578
$Trans_{x}$	0.0101	0.0089	3.1751	3.4187
$Trans_{y}$	0.0077	0.0220	1.9044	3.8176
$Trans_{z}$	0.0054	0.0075	1.6014	2.7850
Total	0.0133	0.0290	2.5977	5.7731
